# Benzofuroxan derivatives N-Br and N-I induce intrinsic apoptosis in melanoma cells by regulating AKT/BIM signaling and display anti metastatic activity in vivo

**DOI:** 10.1186/s12885-015-1835-3

**Published:** 2015-10-27

**Authors:** C. F. Farias, M. H. Massaoka, N. Girola, R. A. Azevedo, A. K. Ferreira, S. D. Jorge, L. C. Tavares, C. R. Figueiredo, L. R. Travassos

**Affiliations:** 1 Experimental Oncology Unit (UNONEX), Department of Microbiology, Immunology and Parasitology, Federal University of São Paulo, Rua Botucatu 862, 8 andar, São Paulo, SP 04023-062 Brazil; 2 Laboratory of Tumor Immunology, Department of Immunology, Institute of Biomedical Sciences, University of São Paulo, São Paulo, SP Brazil; 3 Experimental Physiopathology, Faculty of Medicine, University of São Paulo, São Paulo, SP Brazil; 4 Department of Biochemical and Pharmaceutical Technology, University of São Paulo, São Paulo, SP Brazil

**Keywords:** Melanoma, Benzofuran, Nifuroxazide, Cancer, ROS, Apoptosis

## Abstract

**Background:**

Malignant melanoma is an aggressive type of skin cancer, and despite recent advances in treatment, the survival rate of the metastatic form remains low. Nifuroxazide analogues are drugs based on the substitution of the nitrofuran group by benzofuroxan, in view of the pharmacophore similarity of the nitro group, improving bioavailability, with higher intrinsic activity and less toxicity. Benzofuroxan activity involves the intracellular production of free-radical species. In the present work, we evaluated the antitumor effects of different benzofuroxan derivatives in a murine melanoma model.

**Methods:**

B16F10-Nex2 melanoma cells were used to investigate the antitumor effects of Benzofuroxan derivatives in vitro and in a syngeneic melanoma model in C57Bl/6 mice. Cytotoxicity, morphological changes and reactive oxygen species (ROS) were assessed by a diphenyltetrasolium reagent, optical and fluorescence microscopy, respectively. Annexin-V binding and mitochondrial integrity were analyzed by flow cytometry. Western blotting and colorimetry identified cell signaling proteins.

**Results:**

Benzofuroxan N-Br and N-I derivatives were active against murine and human tumor cell lines, exerting significant protection against metastatic melanoma in a syngeneic model. N-Br and N-I induce apoptosis in melanoma cells, evidenced by specific morphological changes, DNA condensation and degradation, and phosphatidylserine translocation in the plasma membrane. The intrinsic mitochondrial pathway in B16F10-Nex2 cells is suggested owing to reduced outer membrane potential in mitochondria, followed by caspase −9, −3 activation and cleavage of PARP. The cytotoxicity of N-Br and N-I in B16F10-Nex2 cells is mediated by the generation of ROS, inhibited by pre-incubation of the cells with N-acetylcysteine (NAC). The induction of ROS by N-Br and N-I resulted in the inhibition of AKT activation, an important molecule related to tumor cell survival, followed by upregulation of BIM.

**Conclusion:**

We conclude that N-Br and N-I are promising agents aiming at cancer treatment. They may be useful in melanoma therapy as inducers of intrinsic apoptosis and by exerting significant antitumor activity against metastatic melanoma, as presently shown in syngeneic mice.

## Background

Nifuroxazide is a nitroheterocyclic drug belonging to the 5-nitrofuran’s family and used in the treatment of infectious diseases with frequent symptoms of diarrhea and colitis, caused by Salmonella spp, Escherichia coli and Klebsiella [1]. 5-Nitrofurans although being constituents of widely used medications, may cause toxic side effects such as polyneuropathy, depression, forgetfulness, alcohol intolerance, headaches as well as gastrointestinal complications, which interfere with their use [2]. In this context, nifuroxazide analogues were developed replacing the nitrofuran group by benzofuroxan based on the pharmacophore similarity attributed to the nitro group, and also to improve bioavailability, with higher intrinsic activity and less toxicity [2]. Benzofuroxan as well as 5-nitrofurans are used as antibacterial and antiparasitic drugs, giving rise to free radical species [3]. The use of antitumor agents that elevate ROS generation has been effective in many cases with low resistance to these agents [4].

Melanoma is a deadly type of cancer that results from the malignant transformation and uncontrolled proliferation of melanocytes. It is the most invasive skin cancer with increasing incidence and poor prognosis in the metastatic stage [5, 6]. Melanoma of the skin has an incidence of 25,2 % per 100.000 individuals with the US mortality of 3.1 % [7, 8]. Efficient treatment requires early diagnosis, because of the poor prognosis of the disease, especially when it reaches the metastatic stage [9]. With the recent advances in treatment, the 5-year survival rate in melanoma went up to 91 % although that of metastatic melanoma is still averaging 15–20 % of patients. Novel therapeutic approaches are still needed, especially for the metastatic form.

Cancer cells frequently exhibit changes in the redox status associated with increased basal production of ROS, and thus cannot tolerate further intracellular increase of free radicals [4]. Drugs that interfere in redox changes are, therefore, a promising strategy to overcome cancer cell resistance to chemotherapy. Redox status changes may, indeed, exert a critical impact on necrotic/apoptotic and normal cellular processes.

In the present study, we explored the capacity of nifuroxazide analogues to induce ROS-mediated apoptosis in B16F10-Nex2 murine melanoma cells. The signaling pathway affected by the benzofuroxan compounds showed downregulation of Akt and over-expression of pro-apoptotic BIM. A therapeutic protective effect of these compounds in a melanoma syngeneic model is also reported.

## Methods

### Chemicals

The benzofuroxan drugs were synthetized at the Biochemical and Pharmaceutical Technology Department, University of São Paulo, as described [10, 11]. The structure and the substituent groups are indicated below (Table 1). Stock solutions were prepared in DMSO (Sigma-Aldrich, St. Louis, USA) at 10 mM and stored in aliquots at −80 °C. For in vitro tumor cell treatment the stock solution was diluted in RPMI (Sigma-Aldrich, St. Louis, USA), and in PBS 15 % Ethanol (Merck) for in vivo experiments.

**Table 1:** Benzofuroxan compounds and derivatives


Compound	R_1_	R_2_	R_3_	Compound	R_1_	R_2_	R_3_
*1*	H	H	H	*13*	N(CH_3_)_2_	H	H
*2*	CH_3_	H	H	*14*	n-C_4_H_9_	H	H
*3*	NH_2_	H	H	*15*	tert-C_4_H_9_	H	H
*4*	OH	H	H	*16*	OC_3_H_7_	H	H
*5*	F	H	H	*17*	Cl	H	Cl
*6*	CN	H	H	*18*	Cl	Cl	H
*7*	CH_2_CH_3_	H	H	*19*	CF_3_	H	H
*8*	OCH_3_	H	H	*20*	OC_4_H_9_	H	H
*9*	Cl	H	H	*21*	Br	H	H
*10*	COCH_3_	H	H	*22*	SO_2_NH_2_	H	H
*11*	n-C_3_H_7_	H	H	*23*	I	H	H
*12*	Iso-C_3_H_7_	H	H				

### Cell lines

The B16F10 murine melanoma cell line is syngeneic in C57Bl/6 mice and was originally obtained from the Ludwig Institute for Cancer Research (LICR), São Paulo branch. The melanotic subline Nex2 (B16F10-Nex2) was isolated at the Experimental Oncology Unit (UNONEX) and deposited at Banco de Células do Rio de Janeiro (BCRJ) no. 0342. Human cervical carcinoma (HeLa), and human ovarian cancer (Ovcar-3) and the glioblastoma cell line (U87) were provided by Drs. Hugo P. Monteiro, UNIFESP and Osvaldo K. Okamoto, University of São Paulo, respectively. Human colon carcinoma (HCT) was from LICR and mouse colon carcinoma (CT26) was a gift from Prof. Guillermo Mazzolini, Austral University, Argentina.

Tumor cell lineages were maintained in RPMI 1640, pH 7.2, supplemented with 10 mM N-2-hydroxyethylpiperazine-N′-2-ethanesulfonic acid (HEPES), 24 mM sodium bicarbonate, 10 % heat-inactivated fetal bovine serum (FBS) from Gibco (Minneapolis, MN, USA) and 40 μg/ml gentamicin sulfate (Hipolabor Farmacêutica, Sabará, MG, Brazil). Human fibroblasts (GM637 and Fibro T75) were provided by Dr. Luis F. Lima Reis of Sirio-Libanez Hospital, São Paulo, and were grown in DMEM supplemented with 10 mM HEPES, 24 mM sodium bicarbonate, 10 % FBS, and 40 μg/ml gentamicin sulfate.

### Cell viability assay

Viable cells were assessed using the 3-[4,5-dimethylthiazol-2-yl]-2,5-diphenyltetrazolium bromide (MTT) (Sigma-Aldrich, St. Louis, MO) assay. Melanoma cell line B16F10-Nex2 (1.0 × 10^4^ cells/well) were seeded on 96-well plates in RPMI supplemented with 10 % FBS, and compounds (1.5, 3.12, 6.25, 12.5, 25.0, 50.0 or 100 μM) or fresh medium (for control cells) was added. After 16 h, MTT solution (5 mg/ml) in 1x phosphate-buffered saline (PBS) was directly added to the cells, followed by incubation for 4 h at 37 °C. Absorbance was measured at 570 nm with an automated spectrophotometric plate reader (SpectraMax-M2, Molecular Devices Software Pro 5.4, Sunnyvale, CA). The experiments were performed in triplicate. Cell viability was expressed as percent values in comparison with untreated cells.

### DNA degradation

B16F10-Nex2 cells (5 × 10^5^ cells/well) cultured on 6-well plates, were treated with benzofuroxan derivatives N-Br/N-I at IC_50_ and at 100 μM or left untreated at 37 °C for 24 h. Cells were lysed in TELT buffer (50 mM Tris–HCl pH 8.0, Triton X-100 0.4 %, 2.5 mM EDTA pH 9.0, and 2.5 M LiCl). The lysate was centrifuged (12,000 g) for 20 min at 4 °C. Buffer-equilibrated phenol was added (1:1, v/v), followed by centrifugation and addition of chloroform (1:1, v/v). After centrifugation (15 min; 12,000 *g*; 4 °C), the aqueous phase was precipitated with sodium acetate 3 M, pH 7.0 and absolute ethanol (1:0.1:2.5, v/v/v) after overnight incubation at −80 °C. Precipitated DNA was pelleted and diluted in 50 mg/ml of RNAse-A (Invitrogen, Carlsbad, CA). The extracted DNA was subjected to electrophoresis on 1 % Agarose gel with ethidium bromide (0.5 mg/ml) in TBE buffer (2 mM EDTA, 90 mM Tris–HCl, 90 mM boric acid, pH 8.0) at 100 V. One thousand base pair (1 kb) ladder molecular weight markers (Gibco, Grand Island, NY) were used. After electrophoresis, DNA was photographed by UVItec Alliance gel documentation system (UVItec, Cambridge, UK).

### N-acetylcysteine (NAC) effects

B16F10-Nex2 melanoma cells (1.0 × 10^4^/well) were seeded on 96-well plates in RPMI and 10 % FBS, and derivative compounds at 1.5, 3.12, 6.25, 12.5, 25.0, 50.0, or 100 μM were added simultaneously with NAC at 5 mM for 16 h. After incubation, MTT (5 mg/ml) in 1x phosphate-buffered saline (PBS) was directly added to the cells, followed by incubation for 4 h at 37 °C. Absorbance was measured spectrophotometrically (SpectraMax-M2, Molecular Devices Software Pro 5.4, Sunnyvale, CA) at 570 nm. Cell viability was expressed as percent values in comparison to untreated cells.

### ROS determination

Superoxide anions were assessed by dihydroethidium (DHE) assay performed according to manufacturer’s instructions (Invitrogen, Carlsbad, CA). Briefly, 1.0 × 10^4^cells were cultivated on Hi-Q4 dish and treated with N-Br/N-I at IC_50_ for 6 h at 37 °C. Analysis by fluorescence microscopy was carried out in Nikon BioStation IM microscope. Images were processed with ImageJ (http://rsb.info.nih.gov/ij/).

### Annexin V and propidium iodide labeling

B16F10-Nex2 cells (5 × 10^5^/well) were grown in 6-well plates and further incubated with N-Br/N-I at IC_50_ and at 100 μM, or with RPMI 10 % FBS (control) for 24 h at 37 °C. Treated and untreated cells were washed three times with PBS and harvested with a cell scraper. Apoptotic/necrotic cells were detected using the Annexin V-FITC Apoptosis Detection Kit (Sigma-Aldrich, St. Louis, MO). Cells were incubated with binding buffer (10 mM HEPES/NaOH, pH 7.5, 140 mM NaCl and 2.5 mM CaCl2) in presence of propidium iodide (PI) and FITC-labeled annexin V (AV) for 10 min at room temperature and analyzed by flow cytometry (BD Bioscience FACSCanto II, Franklin Lakes, NJ), using FlowJo software (TreeStar Inc., Ashland, OR).

### Chromatin condensation analysis

Murine melanoma cells (1.0 × 10^4^) were cultivated overnight on Hi-Q4 dish and incubated with N-Br/N-I at IC_50_ for 24 h. They were then stained with 1.25 μM Hoechst 33342 (Invitrogen, Carlsbad, CA) for 10 min, washed with PBS and examined for the state of chromatin. Analysis by time-lapsed fluorescence microscopy was carried out on a Nikon BioStation IM microscope. Images were processed with ImageJ (http://rsb.info.nih.gov/ij/).

### Mitochondrial membrane potential (Δψm)

The cationic lipophilic dye tetramethylrhodamine ethyl ester (TMRE) enters the cell in the form of an ester that is subsequently hydrolyzed and converted to tetramethylrhodamine, which is reversibly accumulated in the negatively charged mitochondrial matrix depending on Δψm . B16F10-Nex2 cells (1.0 × 10^5^) were cultured in 12-well culture plate and incubated with N-Br/N-I at IC_50_ for 6 h at 37 °C. Cells were washed with PBS and labeled with 20 nM TMRE (Molecular Probes, OR, USA) for 10 min at 37 °C. Cells were detached with PBS/Trypsin/EDTA and the fluorescence was measured on FACSCanto II (BD Bioscience, Franklin Lakes, NJ), using FACSDiva software (BD Bioscience, Franklin, NJ) and analyzed using FlowJo software (TreeStar Inc., Ashland, OR, USA).

### Cell lysate extracts and Western blotting

B16F10-Nex2 cells (1.0 × 10^6^) treated with N-Br/N-I at IC_50_ and 100 μM or with RPMI 10 % FBS (control cells) for 6 h or 16 h, were washed with PBS and lysed for protein extraction with RIPA buffer (50 mM Tris–Cl, pH 7.5, 150 mM NaCl, 1 % Nonidet P-40, 0.5 % sodium deoxycholate, and 0.1 % SDS) supplemented with protease and phosphatase inhibitors (Sigma–Aldrich, St. Louis, MO). Lysates from cells co-incubated with 5 mM NAC and exposed to derivatives at IC_50_ were also obtained and subjected to Western blotting as described below.

Cell lysates were transferred to microcentrifuge tubes, and kept on ice for 1 h with shaking. They were sonicated for 10 min and centrifuged at 14,000 *g* for 15 min. Protein concentration of lysates was determined by BSA quantification assay (Thermo Fisher Scientific, Rockford, IL). Each cell lysate (30 μg protein) was separated by SDS gel electrophoresis and transferred to nitrocellulose membrane (Millipore, Billerica, MA), blocked with TPBS (PBS, 0.05 % Tween-20) and 5 % (w/v) skim milk or BSA, washed in TPBS and incubated with primary antibody for 16 h on 4 °C. Immunoblotting was run with antibodies against Akt, phospho-Akt (thr308), BIM, caspase 9, caspase 9-cleaved, PARP, PARP-cleaved and β-actin, all purchased from Cell Signaling Technology (Beverly, MA). After washing, membranes were incubated with anti-IgG antibody conjugated with horseradish peroxidase and the immunoreactive bands were detected using Immobilon (Millipore, Billerica, MA, USA) under a chemiluminescence detection system (UVItec, Cambridge, UK). β-Actin was used as loading control.

### Detection of caspase-3 activity

Active caspase 3 was determined in B16F10-Nex2 cells (2.5 × 10^6^ cells) grown with N-Br/N-I at IC_50_, or RPMI 10 % FBS (control) for 8 h. After incubation, cells were tested with Caspase-3 Assay Kit, Colorimetric (Sigma-Aldrich, St. Louis, USA) according to manufacturer’s protocol. Briefly, cells were washed twice with PBS and resuspended in 25 μL of chilled Cell lysis Buffer for 20 min on ice. The lysates were centrifuged at 20,000 *g* for 15 min at 4 °C and 5 μL of the aqueous phase was incubated with 85 μL Assay Buffer and 10 μL caspase-3 substrate Ac-DEVD-pNA 2 mM at 37 °C for 16 h in a 96-well plate. Absorbance was read at 405 nm in a microplate reader (SpectraMax-M2, Software Pro5.4, Molecular Devices, Sunnyvale, CA).

### Experimental melanoma models in vivo

C57BL/6 mice were obtained from the Center for Development of Experimental Models (CEDEME), Federal University of São Paulo (UNIFESP). Animal experiments were approved by the UNIFESP Ethics Committee for Animal Experimentation (CEUA No. 6641300114) based on international recommendations. All in vivo experiments were performed at least twice.

In the lung colonization model, male, six-to-eight week-old, C57BL/6 and NOD/SCID-IL-2R-gamma null mice were challenged endovenously with 5 × 10^5^ syngeneic B16F10-Nex2 melanoma cells in 100 μl of PBS. Animals (*n* = 5 per group) were treated 1 day after tumor challenge, with seven daily intraperitoneal doses of 300 μg (12 mg/Kg) of each benzofuroxan derivative or vehicle (PBS 15 % EtOH). After 14 days, lungs were collected from animals of each group, and the melanotic lung nodules were counted.

### Statistical analysis

Unless otherwise specified, two independent experiments were performed in triplicate. Experimental values are expressed as mean values ± standard deviations (S.D.). For significance analyses Student’s *t*-test was used, GraphPad Prism 4.0 software (La Jolla, CA). *p* values < 0.05 were considered significant.

## Results

### In vitro and in vivo antitumor activity of benzofuroxan compounds

**Fig. 1:**
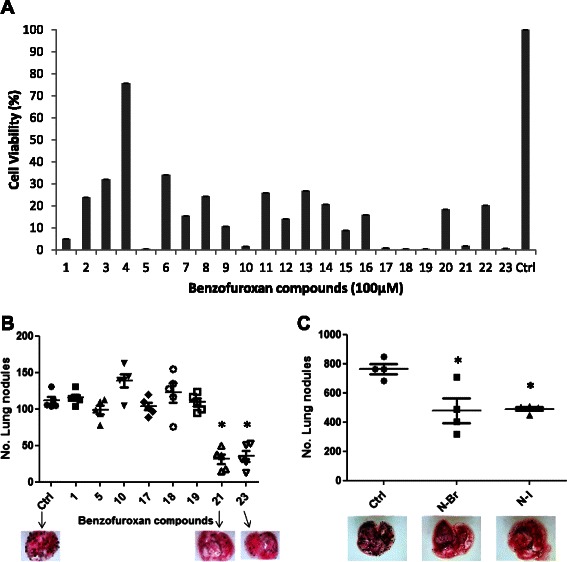
Screening of antitumor activity of benzofuroxan compounds in vitro and in vivo against murine melanoma. **a** Cytotoxicity of 23 compounds in B16F10-Nex2 cells. Compounds were incubated with tumor cells at 100 μM for 16 h and viability was assessed by MTT method. **b** Antitumor activity of active benzofuroxan derivatives in vivo. Number of metastatic melanoma nodules in the lung of animals after treatment with 500 μM of compounds injected i.p. For each experiment, five animals were used per group (*p < 0.05). **c** N-Br and N-I melanoma treatment (300 μg/day) in NOD/SCID IL-2R-gamma null mice for seven consecutive days. No. metastatic nodules were quantified using a Stereo Microscope (Nikon) (*p < 0.05)

The cytotoxicity of the compounds (Table 1) and particularly, the N-Br and N-I derivatives was investigated primarily on murine melanoma B16F10-Nex2 cells at 100 μM for 16 h. All tested compounds were active against tumor cells; compounds 1, 5, 10, 17, 18, 19, 21 and 23 were the most cytotoxic (Fig. 1a). The IC_50_ values (concentrations needed to kill 50 % of tumor cells) were determined on B16F10-Nex2, ranging from 6.9 to 25.4 μM (Table 2). They were evaluated with different murine and human tumor cell lines and non-tumorigenic cells (Table 3). Derivatives N-Br and N-I exhibited similar effects with IC_50_ values between 10 and 30 μM, except for U87 cells, which were resistant to these compounds (IC_50_ > 50 μM).

**Table 2:** IC_50_ (μM) and standard deviations of different benzofuroxan compounds acting on murine melanoma B16F10-Nex2 cells

Compound	IC_50_	SD
1	25.3	±4.3
5	16.7	±2.8
10	25.4	±2.7
17	19.6	±2.0
18	6.9	±0.3
19	9.7	±0.5
21	16.5	±0.9
23	12.0	±0.4

**Table 3:** IC_50_ values obtained for N-Br and N-I treatment of different tumor cells and non-tumorigenic cell lineages

	IC_50_ (μM)	
Cell lineage	N-Br	N-I
B16F10-Nex2	16.5 ± 0.9	12.0 ± 0.4
HCT	32.2 ± 6.6	21.4 ± 3.8
U87	49.1 ± 5.5	58.7 ± 1.4
HeLa	21.7 ± 6.4	25.6 ± 7.2
CT26	34.7 ± 5.8	26.4 ± 4.1
Ovcar-3	18.1 ± 1.9	17.3 ± 3.0
Fibro T75	27.7 ± 2.1	20.6 ± 1.6
GM637	9.4 ± 2.0	9.1 ± 4.3

We also evaluated the antitumor activity of the benzofuroxan compounds in vivo, using a syngeneic lung-colonization (“metastatic”) murine melanoma model. Melanoma cells were injected endovenously in C57Bl/6 mice and treatment was carried out with 7-daily i.p. injections of 300 μg of each compound or vehicle control (PBS 15 % EtOH), starting on day 1 after tumor cell challenge. We observed that only the 21 and 23 derivatives induced significant protection (*p* < 0.05) against metastatic melanoma in vivo (Fig. 1b). Derivatives 21 and 23 (N-Br and N-I), are named 4-Bromo-[*N*′-(benzofuroxan-5-yl)methylene]benzohydrazide (N-Br) and 4-Iodo-[*N*′-(benzofuroxan-5-yl)methylene]benzohydrazide (N-I), respectively.

The effects in C57Bl/6 immunocompetent mice of N-Br and N-I were reproduced also in immune deficient NOD/SCID/IL-2R-gamma^null^ mice [12], which lack T, B and NK cells and circulating complement. As shown in Fig. 1c, both N-Br and N-I significantly reduced the development of pulmonary tumor nodules after 14 days. During the experiment period, all animals maintained healthy physical appearance, normal activity and weight, showing no toxicity of both compounds.

### Morphological changes in melanoma cells treated with N-Br and N-I

**Fig. 2:**
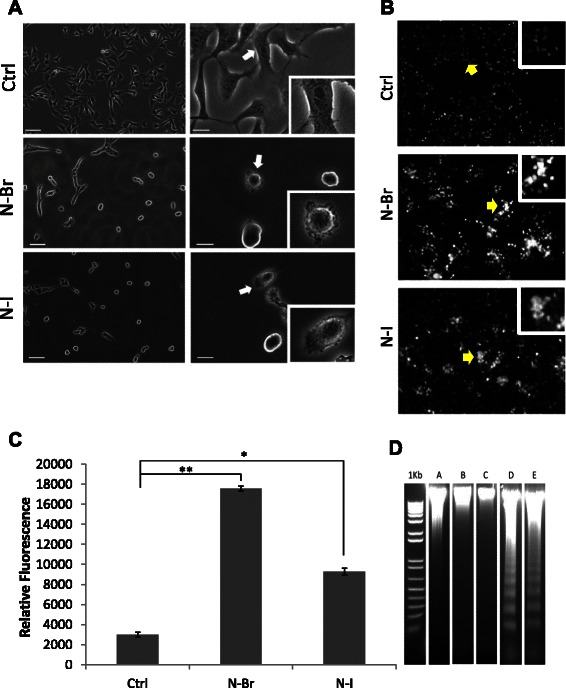
Benzofuroxan derivative effects on melanoma cells (**a**) Morphological effects. Arrows point to surface membrane alterations. Original magnifications, 100× left panels; 200× right panels; 400x inserts. **b** DNA condensation induced by N-Br and N-I. Yellow arrows indicate positive cells for Hoechst staining. 100×, and 400× inserts. **c** Quantification of Hoechst 33342 relative fluorescence in treated and control cells, shown in B; **p ≤ 0.001; *p ≤ 0.05 (**d**) DNA fragmentation showing a ladder pattern; A: Control; B: N-Br (16 μM); C: N-I (12 μM); D: N-Br (100 μM); E: N-I (100 μM)

Both N-Br and N-I caused morphological alterations in B16F10-Nex2 melanoma cells, with cytoplasm retraction, round cells and loss of tumor cell adhesion (Fig. 2a). DNA condensation and fragmentation were also observed in treated cells. Melanoma cells were incubated with both compounds at the IC_50_ concentration for 24 h and the density of nuclear chromatin previously stained with Hoechst was assessed by fluorescence microscopy (Fig. 2b). The relative intensity of fluorescence was quantified by processing images with ImageJ software (Fig. 2c). As to DNA fragmentation, the ladder pattern was observed using Agarose gel electrophoresis, upon treatment with both compounds at 100 μM during 24 h of incubation (Fig. 2d).

### Apoptosis evidence in melanoma cells treated with N-Br and N-I

**Fig. 3:**
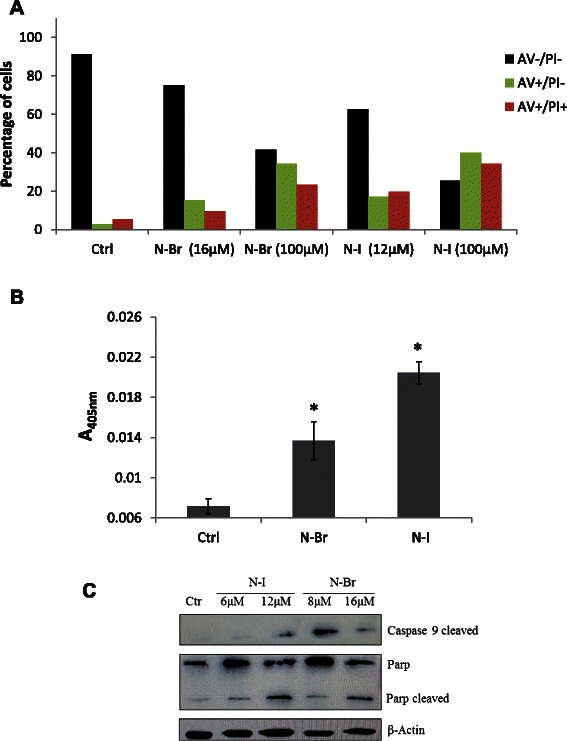
Apoptotic effects of N-Br and N-I in B16F10-Nex2 cells (**a**) Phosphatidylserine translocation determined by annexin V. **b** Caspase-3 activation determined by colorimetric assay (*p < 0,05). **c** Evaluation of caspase-9 and cleavage of PARP induced by the benzofuroxan derivatives

Chromatin condensation and DNA degradation together with phosphatidyl serine translocation to the outer membrane [13] and some characteristic morphological traits (cytoplasm retraction, blebs) are common events of tumor cell apoptosis. We have observed that in addition to the nuclear events, N-Br and N-I cytotoxicity involved significant levels of dose dependent phosphatidylserine accumulation on the outer membrane of B16F10-Nex2 cells, as shown by annexin-V cytometry (Fig. 3a). The number of early apoptotic cells (annexin V^+^/PI^−^) increased as compared to untreated control cells. The treatment with N-Br and N-I at IC_50_ concentration (16 μM for N-Br and 12 μM for N-I) induced apoptosis in 15.2 and 17.2 % of tumor cells respectively. When tested at 100 μM the apoptosis ratio increased to 34.3 % for N-Br and 39.9 % for N-I. Negative control cells showed a very low ratio of annexin-V positive cells (2.8 %).

The role of caspases in tumor cell death by apoptosis is well known, although there are also reports of caspase independent apoptosis [13]. Caspase-3 can be activated in both the intrinsic and extrinsic apoptotic pathways [14]. After treatment of B16F10-Nex2 cells for 6 h with N-Br and N-I, the levels of caspase-3 increased 2 and 2.4-fold as compared to untreated cells (Fig. 3b).

The involvement of other caspases was investigated by Western blotting and both N-Br and N-I activated caspase-9. Moreover, the cleavage of PARP was also observed in treated cells (Fig. 3c).

### N-Br and N-I induce tumor cell death through oxidative stress followed by disruption of ∆ψm

**Fig. 4:**
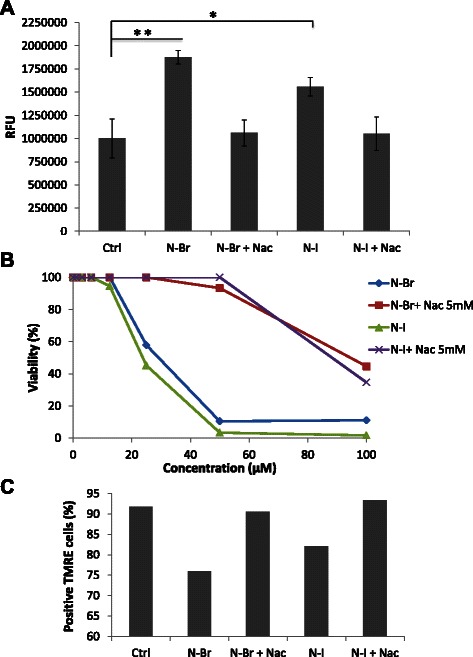
ROS generation and N-acetyl cysteine protective effects. **a** ROS generation after treatment of melanoma cells with N-Br, N-I and NAC, as detected by DHE fluorescence microscopy. **p ≤ 0.001; *p ≤ 0.05 (**b**) Role of ROS on the induction of apoptosis in B16F10-Nex2 cells by the benzofuroxan derivatives and protection by NAC. Cellular viability was assessed by MTT assay. **c** Evaluation of ∆ψm as affected by N-Br and N-I treatment. ΔΨm was assessed using TMRE by flow cytometry

ROS may damage cellular components and trigger a cascade of events leading to cell death [15]. Previous studies with benxofuroxan compounds have shown that these molecules are able to elicit ROS dependent oxidative stress [16]. B16F10-Nex2 cells treated for 6 h with IC_50_ concentrations of the benzofuroxan compounds (16 μM for N-Br and 12 μM for N-I), in presence or absence of NAC, were examined for intracellular ROS revealed with DHE probe. Both compounds led to increased ROS formation evaluated by fluorescence microscopy, as compared to untreated cells, and this effect was attenuated by NAC (Fig. 4a). ROS dependent cell death was further confirmed in a cytotoxicity assay of melanoma cells and the cytotoxic effects of N-Br and N-I were significantly inhibited by NAC (Fig. 4b).

The integrity of ΔΨm, tested by using the lipophilic cationic dye TMRE, was examined in cells treated with the benzofuroxan derivatives [17]. N-Br and N-I significantly decreased the intensity of mitochondrial fluorescence in 15,8 and 9,7 % respectively, indicating that both compounds elicited dissipation of ΔΨm after 6 h of treatment. When tumor cells were incubated with NAC, mitochondria were protected from the cytotoxic effects of both compounds (Fig. 4c).

### N-Br and N-I exert down regulation of AKT and upregulation of BIM in melanoma cells

**Fig. 5:**
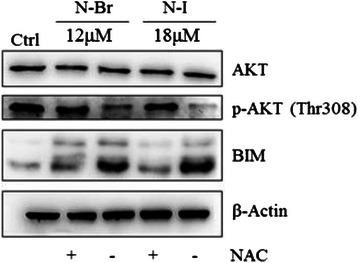
Expression of p-AKT and BIM in B16F10-Nex2 cells treated with N-I and N-Br, assessed by Western blotting. B16F10-Nex2 were treated with both compounds at IC_50_ for 6 h in presence or absence of NAC. Β-actin was used as loading control

AKT activation is involved in the tumorigenesis and resistance to conventional anticancer therapies [18]. In melanoma cells treated with N-Br and N-I, phosphorylation of Akt at Thr380 was reduced, as seen in Western blotting (Fig. 5). Down regulation of active Akt is accompanied by the expression of genes regulating apoptosis. One of these, BIM, encodes a member of “BH3-only proteins”, an important group of pro-apoptotic molecules responsible for mitochondrial stress-induced apoptosis [19, 20]. BIM levels increased in melanoma cells after 6 h treatment with 16 μM of N-Br and 12 μM of N-I (Fig. 5). Indirectly, the involvement of ROS on BIM expression was evaluated by co-incubation of B16F10-Nex2 cells with NAC (5 mM). The reducing activity of NAC restored Akt activity and the expression of BIM was not detected. Therefore, this result shows that the modulation of AKT/BIM signaling depends on ROS generation during the treatment of B16F10-Nex2 cells with the benzofuroxan compounds.

## Discussion

In the current study, we report on the promising antitumor effects of benxofuroxan compounds, which have been shown to induce apoptosis in B16F10-Nex2 melanoma cells. The antibacterial, antiprotozoan and anticancer effects of nifuroxazide related drugs [1], depend on ROS generation [21], and in the present work, we evaluated the antitumor effects of 23 compounds that have the nitrofuran system replaced by a benzofuroxan molecule.

All 23 benzofuroxan compounds exhibited significant levels of cytotoxicity against murine melanoma B16F10-Nex2 cells, and two of them, N-Br and N-I, are the most promising derivatives with antitumor activity in vitro and in vivo. N-Br and N-I exhibited potent cytotoxic activity not only in murine melanoma, but also against several other murine and human tumor cell lines, demonstrating a therapeutic potential in different types of cancers. There was little difference in the IC_50_ values in tumor cells and non-tumorigenic cells, including melan-a (unpublished results), but primary normal cells have not been tested. Cultured cells in the laboratory differ from cells in a living organism in many aspects. Both compounds did not exhibit toxicity in vivo at the concentrations used and yet showed definite antitumor effects in syngeneic mice.

Both N-Br and N-I compounds induced several effects on melanoma cells, related to apoptosis, such as rounding-up, reduction of cellular and nuclear volume, chromatin condensation and DNA fragmentation, and phosphatidylserine outer membrane expression [22, 23]. The loss of ΔΨm, activation of caspase 9 and 3, followed by cleavage of PARP are characteristics of intrinsic apoptosis [24], and were observed upon incubation of N-Br and N-I with melanoma cells.

Given the nature of the benzofuroxan compounds, they increased the levels of ROS in tumor cells [3]. Since co-incubation with the antioxidant N-acetyl cysteine inhibited ROS generation, benzofuroxan drug cytotoxicity and the loss of ΔΨm, we conclude that production of ROS induced by both N-Br and N-I derivatives is involved in their antitumor activity in B16F10-Nex2 cells. Due to their reactive potential, ROS can oxidize DNA bases, leading to DNA strand breaks, intra-strand adducts, crosslinks and mutations [25]. ROS can affect the integrity of proteins and/or lipids in membranes, decreasing the fluidity and increasing membrane permeability as in mitochondria [4]. Mitochondrial dysfunction, characterized by loss of transmembrane potential and opening of mitochondrial permeability transition pores occurs via many apoptotic stimuli [22]. The benzofuroxan derivatives caused disruption of ΔΨmin melanoma cells after 6 h incubation. Impairment of mitochondrial membrane permits the release of cytochrome c, an event known to occur after externalization of phosphatidylserine [23], triggering the apoptotic intrinsic pathway with activation of caspase 9 and 3 [26]. As shown, the apoptotic effects of both benzofuroxan derivatives involved caspase activation.

In addition, N-Br and N-I compounds induced Akt inactivation and expression of pro-apoptotic BH3 protein BIM. Akt is a serine/threonine protein kinase downstream target of the phosphatidylinositol 3-kinase (PI3K) signaling pathway, a central player in response to growth factors or insulin and contribute to several cellular functions including nutrient metabolism, cell growth, transcriptional regulation and cell survival [27]. In a wide variety of cancers, Akt is frequently over activated, contributing to malignancy and related with tumor aggressiveness [18]. Phosphorylation of Akt at Thr308 is essential for Akt catalytic activity [28]. In melanoma, Akt promotes tumor progression, escape from apoptosis and enhanced survival [29]. Recent works have reported that oxidative stress can modulate Akt activation [29–31]. Luo et al. reported that enhanced ROS interact with AKT/FoxO3a/BIM causing inhibition of Akt and consequently nuclear accumulation of FoxO3a, thus facilitating transcription of the target gene BIM [32].

Our results contribute to elucidate the link between Akt signaling and ROS generation. Immunoblotting analysis showed a high expression of phospho-Akt in untreated melanoma cells that was down regulated after treatment with both benzofuroxan derivatives. Co-incubation with NAC blocked these effects suggesting that ROS generation induced in melanoma cells acted as a mediator. Another intracellular signaling molecule sensitive to redox modulation was BIM, which belongs to pro-apoptotic BH3-only proteins family. Consistent with Lou et al. findings [32], downregulation of Akt and increased expression of BIM, which binds to the Bcl-2 family members, led to the loss of ΔΨm [24], thus triggering the intrinsic apoptosis in melanoma cells.

## Conclusion

In conclusion, the present work indicates that benzofuroxan compounds are promising molecules with significant antitumor activities in vitro and in vivo, which should be considered as new therapeutic agents against malignant melanoma.

## Abbreviations

ROS: Reactive oxygen species
NAC: N-Acetylcysteine
Δψm: Mitochondrial membrane potential
TMRE: Tetramethylrhodamine ethyl ester
MTT: 3-[4,5-dimethylthiazol-2-yl]-2,5-diphenyltetrazolium bromide
DHE: Dihydroethidium
